# Phagedæna, a New Disease?

**Published:** 1881-07

**Authors:** G. W. Bowler

**Affiliations:** Cincinnati


### PHAGEDÆNA.—A NEW DISEASE?

---

Editor of Journal of Comparative Medicine:

The term used for the disease I am about to describe is about as appropriate as any I have been able to find, and thus it is I use it. The disease to which I refer made its appearance amongst the horses in Cincinnati about the month of March last, and I find, from investigations, that it has also appeared in other Western cities to an alarming extent, and has in a considerable number of instances proved fatal.

It is a very remarkable affection, and at the same time singular, from the fact that it is entirely different to anything I have heretofore seen make its appearance, nor have I ever seen it described by any veterinary author.

When it first appeared in this city it was supposed to be an attack of what is commonly termed "cracked heel," and was in many instances treated as such, and successfully. But in some cases it assumed a malignant form; then simple remedies were entirely useless, and unless the most active measures were resorted to, the animal would certainly die from blood poisoning, as our experience soon taught us.

The manner in which it generally manifested itself, when of a malignant character, was by the animal's first displaying a slight lameness in one leg, which the owner, as a general thing, attributed to a slight sprain, or other injury to the leg, and in most instances the case was treated as such. But on the following day it assumed an entirely different feature. A small ulcer about the size of a pea would be observed between the fetlock joint and the heel, from which issued a thin, bloody discharge; but so slight is it at first, that but little attention would be paid to it, the owners in some instances supposing it to be a scratch from a nail, or some other foreign substance, with which the animal had come in contact accidentally. These little ulcers were generally washed off, and a poultice of some kind applied; but on the following day, on removing the poultice, it would be found that the sore had become considerably enlarged, and emitted an offensive odor. Still, there would not appear to be anything alarming in the case, until, in the next twenty-four hours, the ulcer displayed every evidence of gangrene, and the surrounding tissues commenced to slough away. If this stage of the disease was allowed to pass without receiving the most vigorous treatment, death from blood poisoning generally occurred on the following day. In two horses which I was called upon to treat, the disease began in the manner described, and both cases assumed the malignant form. Both became lame in front, and were confined to the stable in consequence; but on the following day a small ulcer appeared on the heel of each animal. Poultices of flaxseed were applied to each, but on the following morning the ulcers had considerably enlarged, had an offensive odor, and a large portion of the tissue had sloughed out, leaving a gaping, gangrenous, ugly sore. At this time I was called in, and after cleansing the wound thoroughly, and prescribing a carbolic solution as a dressing, left them until the following day. When I next visited the patients, which was on the following morning, I found the sloughing had extended about half way around the leg. In one case a piece of the integument, as large as a man's hand, was hanging loosely, exposing the ligaments and tendons; a dark-colored, bloody discharge was present, whilst the odor emitted was almost unbearable.

At this time the animals appeared to be very uneasy and feverish, lying down and getting up again frequently, appetite entirely suspended in both instances, respiration increased, and pulse quick and wiry. I was now fully convinced that the animals were suffering from blood poisoning, which had been absorbed from the gangrenous ulcer, and so informed the owner. I also gave it as my opinion that both animals would die, but determined to use such measures as the symptoms called for to relieve them. The bowels were much constipated, and the urine scant, so I administered a cathartic and diuretic, also large doses of bromide potas., fomented the leg with warm water, and dressed with carbolic solution. On the following morning the sloughing had extended all the way around the leg, the suspensory ligaments had given way, and the metacarpal bone was resting on the ground. The bowels had not responded to the cathartic, and in about an hour afterward one of the patients died, and in a few hours afterward the other one also; but in the latter case the disease had extended more to the inner structure of the foot, the hoof being ready to slough off.

The stench from the wounds was at this time fearfully bad, so much so, that I found it necessary to have the stable thoroughly cleaned and disinfected.

Three days afterward I was called on to see another case in the same stable, taken with precisely similar symptoms as the former patients. The ulcer had already commenced sloughing, and from it issued a discharge of the same character as in the other cases. I now came to the conclusion that I had an enemy to contend with, in which the ordinary treatment would not be of much avail, and that if it was not checked at once all treatment afterward would be useless. My first object was to open the bowels, and I gave a cathartic with that object in view. I then thoroughly cleansed the wound, and cut away all the loose parts; then saturated the wound with pure carbolic acid, and in about three minutes washed it again with warm water, and again dressed with the pure carbolic acid, covered the wound with cotton, and bandaged. I then had the leg, "as high up as the knee," sponged frequently with a solution of carbolic acid; in the evening again removed the bandage, and found the sloughing had extended for about two inches on one side. I again applied the pure acid, and bandaged as before. On the following day the bowels had responded, and on removing the bandage I found that the sloughing had ceased, the wound had a healthier appearance, and the odor emitted was less offensive. I then cleansed as before, and applied the same dressing. On the following day dressed with a mild solution, and from that time forward the healing process commenced; the animal made a fine recovery, and has been working ever since. Quite a number of animals in this city, suffering from the same disease, I am given to understand, have died. Since treating the two fatal cases, I have attended quite a number of animals affected in like manner, but, under the treatment described, have been successful in every instance.

I am of the opinion that the disease, in a great measure, originates from the extreme cold winter, aggravated by the admixture of salt and snow, the former of which has been freely scattered along the railroad track during the snowy weather, but which the laws of Ohio have not been able to prevent so far.

Efforts will, however, be made to prevent its being used on our streets during the coming winter.

G. W. Bowler, M.D., V.S.

Cincinnati, June 3d, 1881.

